# Effects of structural connectivity on fine scale population genetic structure of muskrat, *Ondatra zibethicus*

**DOI:** 10.1002/ece3.741

**Published:** 2013-08-29

**Authors:** Sophie Laurence, Matthew J Smith, Albrecht I Schulte-Hostedde

**Affiliations:** 1 Department of Biology, Laurentian University935 Ramsey Lake Road, Sudbury, Ontario, P3E 2C6, Canada; 2 Fundy National ParkP.O. Box 1001, Alma, New Brunswick, E4H 1B4, Canada

**Keywords:** Dispersal, gene flow, landscape genetics, least cost path, microsatellite loci

## Abstract

In heterogeneous landscapes, physical barriers and loss of structural connectivity have been shown to reduce gene flow and therefore lead to population structuring. In this study, we assessed the influence of landscape features on population genetic structure and gene flow of a semiaquatic species, the muskrat. A total of 97 muskrats were sampled from three watersheds near Sudbury, Ontario, Canada. We estimated population genetic structure using 11 microsatellite loci and identified a single genetic cluster and no genetic differences were found among the watersheds as a result of high levels of gene flow. At finer scales, we assessed the correlation between individual pairwise genetic distances and Euclidean distance as well as different models of least cost path (LCP). We used a range of cost values for the landscape types in order to build our LCP models. We found a positive relationship between genetic distance and least cost distance when we considered roads as corridors for movements. Open landscapes and urban areas seemed to restrict but not prevent gene flow within the study area. Our study underlines the high-dispersal ability of generalist species in their use of landscape and highlights how landscape features often considered barriers to animal movements are corridors for other species.

## Introduction

Contemporary population structure can be affected by ecological barriers and decreased structural connectivity between optimal habitat patches (Coulon et al. 2004; Storfer et al. 2007). Recent events including anthropogenic activities and urban development may increase the loss of optimal habitat and decrease connectivity between populations (Cushman 2006; Riley et al. 2006) ultimately resulting in geographical isolation (Trizio et al. 2005; Vandergast et al. 2007). However, Crispo et al. (2011) underlined the positive effects of human activities on gene flow for several species. Anthropogenic features such as roads have usually been shown to negatively affect dispersal and gene flow (Holderegger and Di Giulio 2010). However, some studies have reported positive effects of roads acting as corridors to animal movement (Holderegger and Di Giulio 2010; Crispo et al. 2011). This emphasizes the importance of understanding patterns of gene flow, particularly in complex environments. Features that are a barrier to animal movements in some species may facilitate gene flow in others. The restriction to a specific environment, such as the aquatic environment, increases the effects of barriers on gene flow and therefore can lead to substantial genetic structure (Mullen et al. 2010; Mikulíček and Pišút 2012). The degree of dependence on the aquatic environment varies among semiaquatic species and the terrestrial connectivity between these aquatic habitats is critical for dispersal movements and hence gene flow (Carranza et al. 2012).

Assessing the relationship between gene flow and landscape can be performed by developing a cost surface. Methods used to characterize landscape costs in order to measure resistance surfaces are developing rapidly (Sawyer et al. 2011). Whether using a least cost path (LCP) model or circuit theory (McRae et al. 2008), cost values have to be attributed to the different landscape features. The choice of these cost surfaces is species specific and usually subjective as it is often based on expert opinion (Rayfield et al. 2010; Koen et al. 2012). The assignment of cost surfaces remains one of the challenges of the assessment of functional connectivity, especially in light of the fact that the location of the LCP is sensitive to the relative cost values (Rayfield et al. 2010), and the accumulated cost of the LCP (i.e., the cost distance) increases linearly with increasing relative cost weights (Koen et al. 2012).

Other factors that determine the effect of landscape on population genetic structure are closely linked to the dispersal ability and movement behavior of a species (Clark et al. 2008; Cushman and Lewis 2010). Species with relatively high dispersal ability may present population genetic structure at fine spatial scales due to the influence of landscape structure (Booth et al. 2009; Neaves et al. 2009). High vagility during natal and/or breeding dispersal may enhance gene flow, whereas strong philopatry may decrease it (Temple et al. 2006; Ortego et al. 2011), thus increasing genetic differentiation and population genetic structure. Dispersal behaviors differ among species and vary depending on social structure and mating system (Lawson Handley and Perrin 2007). Differences between the sexes in dispersal have also been shown to affect the genetic structure of populations (Nussey et al. 2005; Chambers and Garant 2011).

We analyzed gene flow in muskrat (*Ondatra zibethicus*) and assessed the effect of landscape features on population genetic structure. This semiaquatic rodent is widespread across North America and uses a wide range of freshwater habitats such as streams, marshes and lakes (Boutin and Birkenholz 1987). Muskrats are dependent on the hydrographic network for shelter, food resources, and reproduction (Boutin and Birkenholz 1987; Ahlers et al. 2010), but they also have the capacity to use terrestrial pathways during dispersal (Errington 1963). Very little is known about the movement abilities of muskrat over different types of terrain and what types of features are considered barriers to movements. Muskrats have small home ranges: approximately 100 m in diameter (Boutin and Birkenholz 1987; Caley 1987) or 582 m in length in linear habitats (Ahlers et al. 2010). They also have limited natal dispersal (<100 m on average) (Caley 1987) as well as limited adult dispersal (30–5 km on average; Errington 1963) during the breeding season or when disturbances occur in the environment such as drought or freezing (Boutin and Birkenholz 1987). Dispersal has been reported as male-biased in muskrat populations (Caley 1987) but it may vary depending on the social structure and mating system (Lawson Handley and Perrin 2007).

We examined the effect of landscape features on the population genetic structure of semiaquatic muskrat using varied cost surfaces to characterize the landscape in order to take into account the path's sensitivity to the cost values and to assess their respective effect on the relationship between genetic distances and least cost path. Semiaquatic species may display different patterns of population genetic structure than strictly terrestrial or aquatic species, and this can have important consequences for species conservation and management in fragmented landscapes. We hypothesized that at a fine spatial scale, fragmentation limits dispersal of muskrats. We predicted that muskrat should exhibit population genetic structure because of the landscape heterogeneity (aquatic and terrestrial) and the presence of anthropogenic features (e.g., roads). More specifically, because of the biology of the species, we predicted that muskrats will show population genetic structure which will reflect the watershed structure and that genetic differentiation should be greater between than within watersheds.

## Methods

### Sample collection

**Figure 1:**
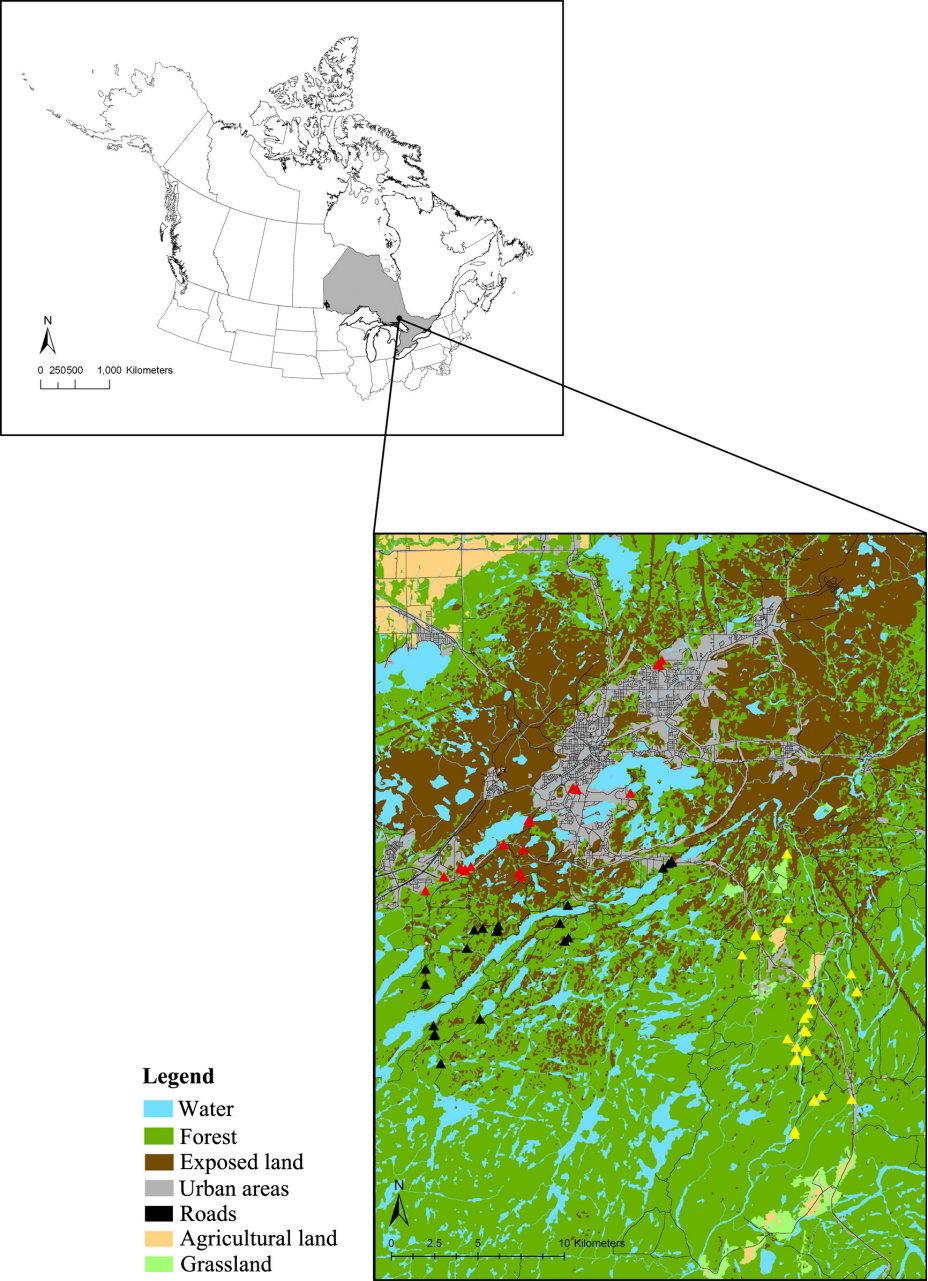
Study area showing the locations of muskrats from the three watersheds: Upper and Lower Junction Creek (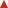), Panache (▲), East Wanapitei (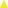) with the landcover types.

Muskrats (*N* = 97) were live trapped in three watersheds located in Sudbury District, Ontario, Canada during May, June and July of 2008 (Fig. 1). Animal trapping and handling was done according to the procedures of the Animal Care Committee of Laurentian University (protocol #2007-04-01) and a Wildlife Scientific Collector's Authorization issued by the Ontario Ministry of Natural Resources (#1039126). Site selection along each watershed was based on accessibility and presence of suitable muskrat habitat. The geographical coordinates of each trap location were recorded using a GPS (Garmin GPSMAP 60Cx; accuracy <10 m). Each individual was marked with an ear tag, weighed, and tissue samples (ear clip) were collected for genetic analysis. We collected 32 samples from the Upper and Lower Junction Creek watershed (Jc), 31 samples from the Panache watershed (Pa) and 34 samples from the East Wanapitei watershed (Wa) (Fig. 1).

Sex determination was performed following the methods described in Ermakov et al. (2006) using the Smc8D and Smc9R primers. Of the 97 individuals sampled, 64 were identified as males and 33 were females. Age was estimated as adult (>1 year old) or juvenile (<1 year old). Because the animals were live trapped and not immobilized, we estimated age using total body mass. Muskrat adult average mass ranges from 900 to 1400 g (Boutin and Birkenholz 1987). In our study, to be conservative, we considered all individuals <1000 g to be juveniles (*n* = 20). The total number of adults was 77, of which 54 were males and 23 were females. Analyses of genetic structure with or without juveniles had no consequences for the resulting output (data not shown); therefore, we maintained all samples in our analyses.

### Genetic analyses

DNA was extracted using QIAGEN DNeasy tissue kits. All individuals were genotyped at 11 microsatellite loci (Oz06, Oz08, Oz16, Oz22, Oz27, Oz32, Oz34, Oz41, Oz43, Oz44, MSCRB5) following Laurence et al. (2009). PCR products were run on an ABI 3730 sequencer and fragment size was determined using Peak Scanner v1.0 (Applied Biosystems, Foster City, CA).

MICRO-CHECKER v2.2.3 (van Oosterhout et al. 2004) was used to test for genotyping errors and for the presence of null alleles, with a confidence interval of 95% and 5000 randomizations. We tested for deviations from Hardy–Weinberg equilibrium (HWE) using GENEPOP version 4.0.7 (Rousset 2008) and linkage disequilibrium (LD) using FSTAT v.2.9.3 (Goudet 2002). To control for multiple tests, sequential Bonferroni corrections (α = 0.05) were used to adjust the level of significance of HWE and LD (Rice 1989).

Genetic diversity was estimated using allelic richness (A), observed (Ho), and expected (He) heterozygosity. Allelic richness was calculated using rarefaction in HP-RARE to control for differences in samples sizes (Kalinowski 2005). Differences in genetic diversity among watersheds were tested using the nonparametric Kruskal–Wallis test (Statistica version 6). Pairwise genetic distance between individuals was determined using the proportion of shared alleles (*D*_ps_) and Rousset's a (*a*_r_) which were calculated using Microsatellite Analyzer (MSA) (Dieringer and Schlötterer 2003) and SPAGeDi 1.3 (Hardy and Vekemans 2002), respectively.

### Effect of sex on dispersal

Sex-biased dispersal was investigated using five different tests across loci: *F*_IS_, *F*_ST_, relatedness, mean assignment index (mAIc), and the variance of these assignment indices (vAIc), implemented in FSTAT v.2.9.3 (Goudet et al. 2002). The dispersing sex will show a higher and positive *F*_IS_, lower *F*_ST_ value, relatedness and mAIc and a higher vAIc than the philopatric sex (Goudet et al. 2002).

We also performed spatial autocorrelation analyses of cumulative distance classes among all trapping locations using the program GENALEX 6.5 (Peakall and Smouse 2012) in order to measure the extent of spatial genetic structure and relatedness for each sex. The analysis was performed for all individuals combined as well as for the sexes separately. We used variable distance classes as the individuals were unevenly distributed across the distances. The distance classes were chosen to maximize the number of pairwise comparisons. However, because of the small sample size of adult females, we were not able to reach 100 pairs as recommended by Hardy and Vekemans (2002). For each distance class, a correlation coefficient was calculated using pairwise genetic distance and geographic distances as implemented in GENALEX 6.5 (Peakall and Smouse 2012). The 95% confidence intervals of the null hypothesis of random distribution were determined using 999 permutations and the 95% confidence intervals for the autocorrelation coefficient were estimated using 1000 bootstraps. The significance of the difference between the sexes for each distance class was also tested using the test for heterogeneity. These sex-biased dispersal analyses were performed on adults only, as they are the individuals of reproductive age and had already dispersed at the time of sampling.

### Population genetic structure

We used several approaches to estimate population genetic structure (Ball et al. 2010; François and Durand 2010). First, we used two individual-based clustering approaches without a priori defined populations: STRUCTURE v. 2.3.1 (Pritchard et al. 2000), a nonspatial Bayesian clustering method, and TESS v.2.3 (Durand et al. 2009) which includes individual spatial information. We ran STRUCTURE with five independent runs per *K*, with *K* ranging from 1 to 10, assuming admixture and correlated alleles. Each run was conducted with a burn-in of 500,000 followed by 500,000 iterations. The most probable *K* was assessed from the posterior probabilities for each value of K (Pritchard et al. 2000) in addition to the Δ*K* (Evanno et al. 2005) as well as from the probability of membership of each individual (q) averaged over the five runs. We ran TESS v.2.3 (Durand et al. 2009) under the assumption of admixture. We ran 50,000 MCMC iterations with 20,000 burn-in for 100 runs, with *K* = 2 to *K* = 10. The most likely *K* was chosen from the deviance information criterion (DIC) values. Second, we also performed a Principal Component Analysis (PCA), a multivariate method adapted to genetic markers, implemented in the Adegenet package in R (Jombart 2008). Pairwise *F*_ST_ values were calculated for the three watersheds using permutations to test for significance as implemented in FSTAT v.2.9.3 (Goudet et al. 2002).

### Least cost path analysis

Landscape data for the Sudbury district were obtained from the National Hydro Network GeoBase (2004) for the water bodies data, Statistics Canada (2006) for the road network and Land Cover Circa GeoBase (2000) for the land cover. All of the landscape characteristics of our study area were aggregated into three or four types of landscape cover that could potentially impact muskrat movement (positively or negatively). We reclassified the landscape types into categories that corresponded to low, medium and high cost for muskrat movements, based on previous studies of muskrat spatial ecology (Errington 1963; Virgl and Messier 1996; Ahlers et al. 2010). Because we did not know the effect of roads on muskrat movements, we built two raster maps. The first one consisted of three categories of landscape: water, forest and “open landscape and human activity”. The category “open landscape and human activity” included grassland, exposed land (i.e., rock outcrop, barren land), agricultural land, roads and urban areas (i.e., residential, commercial, industrial). The second raster map consisted of four categories: water, roads, forest, and “open landscape and human activity” (which combined grassland, exposed land, agricultural land, and urban areas). We also included dams and waterfalls as impermeable barriers. These land cover types were mapped on raster maps with a cell size of 20 m by 20 m.

We allocated resistance values to each cell in order to calculate least cost paths (LCP). However, resistance values are often chosen arbitrarily and the range of these values are also variable (Sawyer et al. 2011). In order to take into account the effect of the values chosen, we built several models with different cost schemes (see Table 3 for examples of models) (Desrochers et al. 2010; Sawyer et al. 2011). Pairwise LCP distances in meters were calculated for each cost model using Pathmatrix 1.1 (Ray 2005) an extension of the geographical information system software ARCVIEW 3.X (Environmental Science Research Institute, Redlands, CA).

We compared pairwise genetic distances to Euclidean distances and the different effective distances (33 LCP models) using partial Mantel tests with 10,000 permutations, which were calculated using the package ecodist version 1.2.7 (Goslee and Urban 2007) implemented in R 2.13.0 (R Development Core Team 2011). We used partial Mantel tests in order to control for the Euclidean distance on the relationships between the genetic distances and the LCP models. *P*-values were adjusted for multiple tests using false discovery rates (FDR) (Pike 2011).

## Results

### Genetic structure

No evidence of genotyping errors was found and two loci (Oz08 and Oz22) were suspected to show presence of null alleles (estimated % of null alleles: 12.1 and 19.1%, respectively). We did not detect LD after Bonferroni corrections and two out of 11 loci were not in HWE after sequential Bonferroni corrections (Oz08 *P* < 0.001 and Oz22 *P* < 0.0001). These two loci were removed from further analyses so all the results presented were obtained using nine microsatellite loci.

Genetic diversity was not significantly different among the three watersheds (Kruskal–Wallis *P* > 0.05) and was highly diverse in all three regions (Table 1). Overall, mean number of alleles per locus (A) was 15.8 (± 5.74) with A ranging from 11.3 to 12.1 (Table 1). Observed and expected heterozygosities for all samples were 0.81 (± 0.11) and 0.83 (± 0.09), respectively (Table 1).

**Table 1:** Genetic diversity of muskrat (*Ondatra zibethicus*) in the three watersheds in the Sudbury District, Ontario. Number of individuals (*N*), allelic richness (A), expected heterozygosity (*H*_e_), observed heterozygosity (*H*_o_) are indicated with standard deviation in brackets

Watershed	*N*	A	*H*_e_	*H*_o_
Junction creek	32	11.8 (4.00)	0.81 (0.11)	0.81 (0.14)
Panache	31	12.1 (4.12)	0.82 (0.08)	0.78 (0.08)
East Wanapitei	34	11.3 (3.73)	0.82 (0.10)	0.85 (0.13)
Total	97	15.8 (5.74)	0.83 (0.09)	0.81 (0.11)

We did not detect any significant sex-biased dispersal (Table 2). However, although not statistically significant, four of the five tests used to examine sex bias in dispersal showed a tendency toward male-biased dispersal with a higher *F*_IS_, lower *F*_ST_ and lower relatedness (Table 2). The mAIc was higher and positive for females (0.581) and negative for males (−0.252), but again not statistically significant. The variance of AIc is expected to be higher in the dispersing sex; however, we found that vAIc was higher in females but not significant, indicating a tendency toward female-biased dispersal (Table 2).

**Table 2:** Results of sex-biased dispersal tests in adult muskrats (*Ondatra zibethicus*)

				Assignment indices	
				
	*F*_IS_	*F*_ST_	Relatedness	Mean	Variance
Females (*n* = 23)	0.041	0.019	0.035	0.581	17.330
Males (*n* = 54)	0.074	0.012	0.022	−0.252	9.239
P values	0.269	0.380	0.370	0.204	0.988
Overall (*n* = 77)	0.068	0.010	0.019	–	–

Significance values were calculated using 5000 permutations.

**Figure 2:**
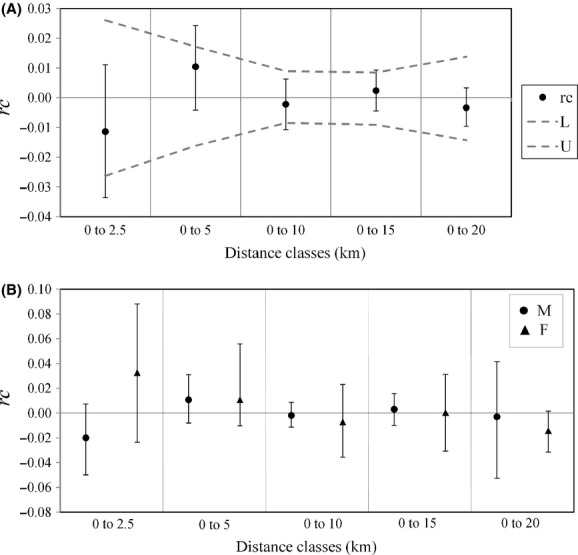
Spatial autocorrelograms of the cumulative distance classes. Correlation coefficients are presented all individuals (A) and males and females (B). The 95% confidence error bars and the permuted 95% confidence intervals (dashed lines) for the null hypothesis of random distribution are presented.

The spatial autocorrelation analyses of cumulative distance classes showed similar patterns of genetic structure in both sexes (Fig. 2B). Both sexes had correlation coefficients not significant from random for any of the distance classes. Females displayed a positive *r*-value within the 0–3 km distance class (*r* = 0.033); however, it was not significant. The test for heterogeneity did not detect a difference in spatial genetic structure patterns between sexes (0.200 ≤ *P* ≥ 0.991) indicating homogeneity between the spatial correlograms.

Both type of analyses indicated a lack of sex-biased dispersal. However, the tests from Goudet et al. (2002) showed an absence of sex-biased dispersal with a tendency toward male-biased dispersal and the spatial autocorrelation analysis indicated a tendency toward female philopatry under 2.5 km. Consequently, the genetic structure analyses were performed for all samples as well as for the sexes separated. We did not detect any differences in the results when separating the sexes and therefore all the results presented henceforth include male and female samples combined. The spatial autocorrelation analysis of cumulative distance classes indicated that individuals *rc* (males and females pooled) were not significantly different from random for all the distance classes (Fig. 2A).

**Figure 3:**
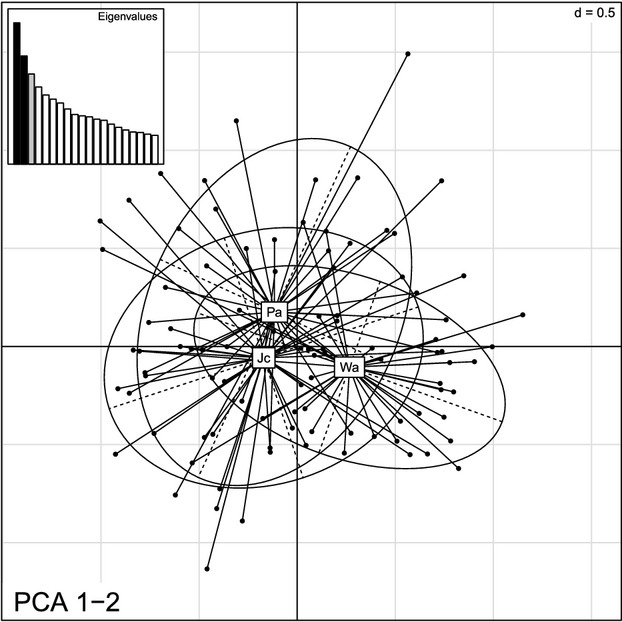
PCA of the first two principal components for the three watersheds: Upper and Lower Junction Creek (Jc), Panache (Pa), and East Wanapitei (Wa).

A single cluster (*K* = 1) was suggested by STRUCTURE. Although the highest LnP(*K*) was detected at *K* = 5 (average LnP(*K*) = −4025), suggesting the presence of five clusters, the proportion of individuals ancestry (q) was low (0.377 ± 0.178). These results indicate that *K* = 5 is not the true *K* and the number of genetic cluster is one. Similar results were obtained using the spatial Bayesian clustering method TESS. Comparable results indicating one cluster were also obtained using the PCA (Fig. 3) with 8.2% of the variance explained by the first axis and 6.3% of the variance explained by the second axis, providing further evidence that muskrats from the three individual watersheds were not genetically distinct. Pairwise *F*_ST_ values were significantly different from zero between East Wanapitei and Junction creek (*F*_ST_ = 0.0174 *P* ≤ 0.05) and between East Wanapitei and Panache (*F*_ST_ = 0.0164 *P* ≤ 0.05) but not between Junction creek and Panache (*F*_ST_ = 0.0001).

### Least cost path analysis

The full Mantel tests performed between the genetic distances (*a*_r_ and *D*_ps_) and the Euclidean distance were significant (*P* = 0.0002 and *P* = 0.0001, respectively), but showed weak Mantel *r* (0.108 and 0.154, respectively; Table 3). After partialling out the Euclidean distance from the LCP models, the only significant relationships after adjusting the *P*-values for multiple tests (0.014 < *P* < 0.012) were found between *D*_ps_ and LCP models that considered the roads as facilitator models for muskrat movements (Table 3).

**Table 3:** Results of partial Mantel tests between the genetic distances (*a*_r_ and *D*_ps_) and the geographic distances in meters: Euclidean distance (first row) and the different LCP models (four categories of landscape). All partial Mantel tests are partialling out the Euclidean distance

Resistance to movement				*a*_r_		*D*_ps_	
		
Water	Roads	Forest	Open landscape + human activity	Partial Mantel's *r* (CI)	*P*	Partial Mantel's *r*	*P*
1	1	1	1	0.108 (0.080 to 0.134)	0.0002	0.154 (0.129 to 0.176)	0.0001
1	1	2	4	−0.026 (−0.054 to 0.005)	0.764	−0.014 (−0.040 to 0.007)	0.681
1	1	5	10	0.018 (−0.013 to 0.049)	0.336	0.053 (0.030 to 0.077)	0.063
1	1	5	50	0.011 (−0.018 to 0.041)	0.390	0.050 (0.025 to 0.073)	0.067
1	1	10	20	0.026 (−0.009 to 0.060)	0.251	0.058 (0.033 to 0.081)	0.037
1	1	10	100	0.029 (−0.0001 to 0.063)	0.218	0.062 (0.037 to 0.084)	0.029
1	1	25	50	0.024 (−0.003 to 0.053)	0.271	0.060 (0.035 to 0.083)	0.033
1	1	50	100	0.035 (−0.0002 to 0.064)	0.190	0.077 (0.052 to 0.102)	0.012*
1	1	50	500	0.035 (0.006 to 0.064)	0.189	0.077 (0.054 to 0.100)	0.012*
1	1	100	200	0.031 (−0.001 to 0.057)	0.223	0.078 (0.055 to 0.101)	0.012*
1	1	100	1000	0.031 (−0.0002 to 0.060)	0.231	0.078 (0.054 to 0.104)	0.011*
1	1	250	500	0.031 (0.002 to 0.059)	0.223	0.078 (0.051 to 0.104)	0.012*
1	1	500	1000	0.030 (0.002 to 0.059)	0.232	0.078 (0.053 to 0.103)	0.014*
1	10	10	20	−0.002 (−0.024 to 0.023)	0.527	0.014 (−0.007 to 0.035)	0.334
1	10	10	100	−0.012 (−0.038 to 0.017)	0.620	−0.007 (−0.030 to 0.014)	0.595
1	10	25	50	−0.020 (−0.043 to 0.005)	0.692	−0.013 (−0.034 to 0.012)	0.657
1	10	50	100	−0.043 (−0.071 to −0.014)	0.842	−0.021 (−0.045 to 0.005)	0.721
1	10	100	200	−0.043 (−0.068 to −0.011)	0.835	−0.030 (−0.053 to −0.001)	0.784
1	10	100	1000	−0.043 (−0.068 to −0.011)	0.846	−0.030 (−0.051 to −0.004)	0.793
1	10	250	500	−0.027 (−0.052 to −0.002)	0.745	−0.007 (−0.029 to 0.018)	0.586
1	10	500	1000	−0.026 (−0.048 to −0.001)	0.740	−0.009 (−0.032 to 0.019)	0.599

95% confidence intervals (CI) are indicated in parentheses.

* Significant result using false discovery rates adjusted *P*-values.

## Discussion

One panmictic population was detected in our study area, and no evidence of genetic differentiation within or among the three watersheds was observed. The population had a high genetic diversity. These results were consistent among the various approaches used to estimate population genetic structure at a fine spatial scale. This continuous distribution of muskrat with no genetic differentiation between watersheds suggests substantial gene flow throughout the study area.

The individual-based approaches (STRUCTURE, TESS, and PCA) did not demonstrate genetic structure at the fine geographical scale. Underlying patterns of isolation by distance can make it difficult to interpret the results from Bayesian clustering methods (Pritchard et al. 2000; Frantz et al. 2009) and these results should be taken with caution. Nevertheless, we also detected one population using the ordination method (PCA). Pairwise *F*_ST_ values indicated significant differentiation between East Wanapitei and the other watersheds. However, using a priori defined populations may lead to significant *F*_ST_ values particularly when an isolation by distance pattern is present (Gauffre et al. 2008; Frantz et al. 2010; Wasserman et al. 2010); which is most likely the case in our study. The spatial autocorrelation analysis of cumulative distance classes did not detect spatial genetic structuring in relation to distance at this fine spatial scale. Although there is limited information regarding the dispersal capacities of muskrats, Errington (1963) reported limited dispersal distances ranging from 30 m to 5 km with the majority of individuals dispersing to a maximum of 100 m. However, muskrats have the ability to travel over long distances particularly in response to extreme conditions such as drought or high population density, with individuals dispersing to up to 34 km (Errington 1963). Artimo (1960) reported dispersal distances of 4–120 km per year in Finland with the majority of individuals dispersing within the distance category of 10–20 km.

We did not detect spatial genetic structuring for both sexes suggesting a lack of sex-biased dispersal at this spatial scale. Dispersal in mammals is usually biased toward males (reviewed by Lawson Handley and Perrin 2007); however, we did not detect clear evidence of sex-biased dispersal in this study as suggested by the spatial autocorrelation analysis that showed similar patterns in both sexes. Although the lack of sex-biased dispersal in mammals has rarely been observed, it has been reported in several mammals such as the European roe deer (*Capreolus capreolus*) (Bonnot et al. 2010) and the southern water vole (*Arvicola sapidus*) (Centeno-Cuadros et al. 2011). Social and mating systems influence sex bias in mammal dispersal (Lawson Handley and Perrin 2007). Polygynous species often display male-biased dispersal, whereas monogamous species display female-biased dispersal; however, this rule does not apply for several species (Lawson Handley and Perrin 2007). Muskrats have been reported to be polygynous in some regions and monogamous in others (Marinelli et al. 1997) suggesting that the mating system alone cannot explain the absence of bias in dispersal in this species. Patterns of breeding dispersal and natal dispersal may also influence dispersal and explain the lack of bias in some species (Coulon et al. 2006). Further study on the social structure of muskrat population is necessary in order to better understand dispersal patterns. We did observe a tendency of female philopatry up to 2.5 km; however, it was not significant. One of the limitations of these results is the small female sample size, which reduces the power of the tests (Lawson Handley and Perrin 2007).

Contrary to our hypothesis, our results showed that population genetic structure was not influenced by the landscape structure at a fine geographical scale. However, genetic distances among individuals were partly explained by landscape features. We detected only one population in our study area and no barriers were identified which seems to support the idea that no landscape features seemed to prevent gene flow. However, when comparing the different models, models with high cost for “open landscape and urban areas” and “forest” were the models with the highest significant partial mantel's r, which seems to indicate that they do prevent, to some extent, gene flow. Open landscape and urban areas and to some extent forest seemed to restrict but not prevent gene flow in muskrat. Muskrat movements are influenced by the landscape, but gene flow was not prevented by the landscape characteristics in our study and no landscape barrier was identified. Although Ahlers et al. (2010) found that muskrat home ranges were linear and restricted to river banks; we did not find that gene flow was limited to the watershed. This linear use of habitat may be explained by the substantial presence of agricultural land in their study sites preventing muskrat movements outside of stream networks. Our study area did not contain a high percentage of agricultural land (approximately 1%), thus allowing individuals to use nonagricultural terrestrial corridors. Our results suggest a limited effect from landscape features and presence of isolation by distance. Gauffre et al. (2008) did not detect any barrier to gene flow in common vole (*Microtus arvalis*) populations. The authors explained this isolation by distance pattern and the lack of effect of landscape fragmentation on gene flow by the high effective population size of this species and the barrier being too recent to affect such a large population.

Stream connectivity has been shown to influence patterns of genetic structure in amphibian species (Mullen et al. 2010). In semiaquatic mammals, this relationship has rarely been demonstrated because of their ability to use terrestrial corridors (Vignieri 2005; Zalewski et al. 2009). Similarly, we did not find that muskrat population structure reflected watershed network, which may highlight the opportunistic behavior of this species in the use of terrestrial landscape for movements. Muskrat may be considered generalist in their use of the landscape for dispersal. Zalewski et al. (2009) showed that population genetic structure of American mink (*Neovison vison*) did not reflect the watershed structure and that gene flow was not influenced by connection between waterways. We observed similar results in muskrats, indicating the high dispersal ability of these semiaquatic species and their capacity to use terrestrial corridors for dispersal. Different dispersal strategies are predicted in generalist species as opposed to specialist ones. Habitat specialists may be more affected by landscape structure than generalists; however, Centeno-Cuadros et al. (2011) have demonstrated that the southern water vole, although considered a habitat specialist, uses a wide variety of landscape types during dispersal, thus increasing gene flow.

Cotner and Schooley (2011) found that muskrat were more abundant in urban areas, and considered the muskrat as an urban adapter. The presence of numerous water bodies (approximately 13.5% of the total area) in the urban areas of our study area may explain the high tolerance of muskrats to human activities. As suggested by Cotner and Schooley (2011), these water bodies could be used for dispersal while rivers could be used for house dwelling providing the presence of vegetation as food resources such as cattail (*Typha* sp.). Most studies report on the negative impact of human activities on dispersal due to landscape fragmentation (Keyghobadi 2007; Magle et al. 2010; Fenderson et al. 2011). However, human activities can also positively affect gene flow by creating corridors associated with roads (Crispo et al. 2011). Our results showed that including roads in the landscape category of facilitator models for muskrat movement improved the relationship of genetic distance with the LCP models. For the muskrat, ditches and culverts may create movement corridors that can be used to connect optimal habitats thus facilitating dispersal and gene flow. Although roads did not seem to act as a barrier in our study, we have not considered traffic patterns. High traffic levels may have a negative impact on gene flow, for example, in increasing mortality (Fahrig and Rytwinski 2009). Human activities may also have a positive impact by reducing the risk of predation as predators are generally more affected by human disturbances (Leighton et al. 2010). Finally, in the case of furbearers such as the muskrat, another potential positive effect of urban areas is the lower trapping pressure (Cotner and Schooley 2011).

We found significant patterns using *D*_ps_ but not *a*_r_. We may have detected significant pattern using *D*_ps_ because it is a genetic distance that does not require equilibrium assumptions (Bowcock et al. 1994). Moreover, it has been shown that *D*_ps_ has the power to detect population genetic structure and connectivity at small spatial scale (Murphy et al. 2010). On the other hand, Rousset's genetic distance (*a*_r_) may be considered as the equivalent of *F*_ST_-based measures and is more appropriate for examining relationships with historical landscape data (Balkenhol et al. 2009). This may explain why it is less likely to detect significant patterns using Rousset's *a*_r_ associated with contemporary landscape data. The choice of resistance cost for the landscape types can affect the results of landscape genetic studies as the relative cost value will change the model sensitivity (Rayfield et al. 2010; Sawyer et al. 2011; Koen et al. 2012). Changing the cost of landscape types did change the least cost path used between individuals but did not change the sensitivity of our relationship. It is possible that our study was conducted at too small a spatial scale to detect differences in genetic structure as the effects of landscape on gene flow are scale dependent. An increase in the size of the study area may allow us to detect the presence of population genetic structure at a larger geographical scale with an effect of landscape characteristics in shaping this structure. The temporal scale may also have an effect on the dynamics of corridors and barriers (Anderson et al. 2010). In several studies, landscape modifications have occurred too recently to affect populations which may explain the lack of detection of population genetic structure (Anderson et al. 2010; Bennett et al. 2010). In our study, the perturbations (urban areas and roads) were well established at the time of study and we do not think that the temporal scale explains the lack of population structure. Future research should increase the spatial scale of study and include other landscape characteristics that may play a role in muskrat dispersal such as substrate composition, bank height, and the width of streams (Cotner and Schooley 2011). Landscape genetic studies of muskrat should also look at the effect of the different types of roads as well as the different traffic levels. We must be careful though not to overstate these results due to our limited sample size (particularly the female sample size), and increasing the number of individuals may help in increasing the power of the results.

In conclusion, population genetic structure of muskrat was not influenced by landscape composition, and landscape features had a limited effect on gene flow. Muskrats had the capacity to use terrestrial pathways between watersheds and roads were not a barrier to movements. On the contrary, it seems that roads may be used as corridors for movements. Our study suggests that semiaquatic species may be less sensitive to landscape fragmentation than species more restricted to aquatic environments such as amphibians.
